# Supplementary material for: Resolving the full spectrum of human genome variation using Linked-Reads
Source: Genome Res. 2019 Apr;29(4):635–45. doi: 10.1101/gr.234443.118 (PMC6442396; doi:10.1101/gr.234443.118)
Supplement: Supplemental Material [file supp_gr.234443.118_Supplemental_longranger-2.2.zip › longranger-2.2.tar.bz2!/lr2.2-public/lib/stan/cmdstan/stan/doc/api/html/validate__non__negative__index_8hpp_source.html]

Stan: src/stan/math/error\_handling/matrix/validate\_non\_negative\_index.hpp Source File


|  |  |
| --- | --- |
|  | Stan  2.5.0  probability, sampling & optimization |


- Main Page
- Related Pages
- Namespaces
- Classes
- Files

- File List
- File Members

All Classes Namespaces Files Functions Variables Typedefs Enumerations Enumerator Friends Macros Pages

- src
- stan
- math
- error\_handling
- matrix

validate\_non\_negative\_index.hpp

Go to the documentation of this file.

1 #ifndef STAN\_\_MATH\_\_ERROR\_HANDLING\_\_MATRIX\_\_VALIDATE\_NON\_NEGATIVE\_INDEX\_HPP

2 #define STAN\_\_MATH\_\_ERROR\_HANDLING\_\_MATRIX\_\_VALIDATE\_NON\_NEGATIVE\_INDEX\_HPP

3

4 #include <sstream>

5 #include <stdexcept>

6

7 namespace stan {

8  namespace math {

9

10  inline

11  void

12  validate\_non\_negative\_index(const std::string& var\_name,

13  const std::string& expr,

14  int val) {

15  if (val < 0) {

16  std::stringstream msg;

17  msg << "Found negative dimension size in variable declaration"

18  << "; variable=" << var\_name

19  << "; dimension size expression=" << expr

20  << "; expression value=" << val;

21  throw std::invalid\_argument(msg.str());

22  }

23  }

24

25  }

26 }

27 #endif

stan::math::validate\_non\_negative\_index

void validate\_non\_negative\_index(const std::string &var\_name, const std::string &expr, int val)

**Definition:** validate\_non\_negative\_index.hpp:12


---

[ Stan Home Page ]

*© 2011–2014,
Stan Development Team.*
